# Chilean *Aloysia* Essential Oils: A Medicinal Plant Resource for Postharvest Disease Control

**DOI:** 10.3390/plants14203121

**Published:** 2025-10-10

**Authors:** Valentina Silva, Catalina Ferreira, Susana Flores, Evelyn Muñoz, Constanza Reyes, Carmen Trujillo, Esperanza Gálvez, Katy Díaz, Alejandro Madrid

**Affiliations:** 1 Laboratorio de Productos Naturales y Síntesis Orgánica (LPNSO), Departamento de Ciencias y Geografía, Facultad de Ciencias Naturales y Exactas, Universidad de Playa Ancha, Avda. Leopoldo Carvallo 270, Playa Ancha, Valparaíso 2340000, Chile; silvapedrerosv@gmail.com (V.S.); catalina.ferreira.fs@gmail.com (C.F.); s.flores.gonzalez@gmail.com (S.F.); evdmunoz@gmail.com (E.M.); reyesveraconstanza@gmail.com (C.R.); ctrujillocadiz@gmail.com (C.T.); esperanza.galvez@alumnos.upla.cl (E.G.); 2 Departamento de Química, Universidad Técnica Federico Santa María, Av. España N° 1680, Valparaíso 2340000, Chile; katy.diaz@usm.cl

**Keywords:** farnesol, biopesticide, phytopathogenic, fruit rot, postharvest decay

## Abstract

Postharvest fungal rot causes significant economic losses in the agroindustry. Current control methods involving the use of synthetic fungicides are becoming increasingly ineffective and pose environmental risks. This necessitates exploring sustainable alternatives, such as essential oils derived from medicinal plants, to achieve safer and effective disease control. This research examined the chemical composition and efficacy of essential oils from *Aloysia citriodora*, *Aloysia polystachya* and their compounds against the postharvest rot fungi *Monilinia fructicola*, *Monilinia laxa*, and *Botrytis cinerea*. The main compounds of essential oils were analyzed by GC/MS and revealed differences in their composition. *A. citriodora* is characterized by the presence of spathulenol and caryophyllene oxide. In contrast, *A. polystachya* is characterized by the predominance of carvone. The results show that the essential oil of *A. citriodora* and the compound farnesol are able to inhibit the three pathogens. Notably, against *M. fructicola*, the EC_50_ values were 61.89 μg/mL and 72.18 μg/mL, respectively. Against *B. cinerea*, the EC_50_ values were 85.34 μg/mL and 47.6 μg/mL. Molecular docking also showed that farnesol has affinity for the enzyme succinate dehydrogenase suggesting a possible mechanism of action. This compound and *A. citriodora* essential oil show potential in the control of phytopathogens.

## 1. Introduction

Phytopathogenic fungi are a major contributor to plant diseases, causing significant economic losses and posing food safety risks due to production and accumulation of mycotoxins. Some disease-causing agents, such as *Monilinia fructicola*, *Monilinia laxa* and *Botrytis cinerea* are classified as necrotrophs, as they feed on dead host tissues. These pathogens are responsible for gray mold and brown rot, correspondingly, culminating in severe fungal ailments [1,2]. The pathogenicity mechanism exhibited by these fungal includes the exudation of toxins, enzymes responsible for the degradation of cellular walls, and other substances unique to the pathogenic agents [3], these fungal species exhibit a global distribution and are acknowledged for their capacity to induce wilting in floral structures, cause canker formation in ligneous tissues, and trigger decay in fruit bodies. Its life cycle depends on conidia, which are spread by wind, insects, or rain. The pathogen’s growth depends on favorable conditions like high humidity, frequent rainfall, and warm temperatures [4,5].

The interest in studying *B. cinerea* arises from the significant losses it causes in crop yield, resulting in economic damage ranging from USD $10 to $100 billion globally [6]. It also affects a wide variety of economically important crops, including strawberries, stone fruits, kiwifruits, tomatoes and grapes, especially in their postharvest [7]. This pathogen is hard to control due to its ability to infect many different plants and its diverse ways of causing disease. As a result, it leads to various symptoms, but the most recognizable one is the development of soft rot accompanied by gray masses of conidia, commonly known as gray mold [4]. Furthermore, approximately 10% of post-harvest productivity worldwide is lost due to brown rot disease caused by *M. fructicola* and *M. laxa*, it is an important constraint, difficult to control, which spreads rapidly in commercial crops in wet seasons especially in its main hosts *Prunus* sp. such as *P. avium* (Cherry), *P. domestica* (Plum), *P. persica* (Peach), *P. nucipersica* (Nectarine), *P. salicina* (Japanese Plum) among others, with emphasis during postharvest when ideal conditions for infection are achieved [8,9].

The European Union authorizes the use of fungicides such as quinone-outside inhibitors (QoI), succinate dehydrogenase inhibitors (SDHI) and methyl benzimidazole carbamates (MBCs) for the control of these phytopathogens [10]; the latter interact with the β-tubulin receptor, altering the formation of microtubules and consequently preventing mitosis in the fungus. The resistance mechanisms reported for these fungicides are related to binding site mutations in β-tubulin proteins for the case of studies conducted on *Monilinia* sp. [11]. On the other hand, monitoring of *B. cinerea* strains in different crops have shown a sustained increase in fungal resistance to these fungicides, including multi-resistance in crops that have been subjected to their use for years [10]. Among other treatments used for brown rot, copper-based products also are an alternative for its control [12]; however, copper is persistent in soil, not biodegradable and is toxic to non-target organisms and aquatic ecosystems, with long-lasting effects [13,14,15]. The economic impact of these phytopathogens in the agricultural industry, the resistance generated to their traditional control, their potential adverse effects on the environment, have led to the search for options for the control of phytopathogenic organisms that affect crops of agricultural interest.

The literature reports the use of essential oils derived from medicinal and aromatic plants as an alternative to control phytopathogens [16]. Essential oils, in general, contain monoterpenes and sesquiterpenes that show different mechanisms of action depending on their origin and composition; they may also have synergistic effects that reduce the risk of resistance development and are therefore considered as ingredients for the development of new biopesticides [17].

*Aloysia citriodora* and *Aloysia polystachya* commonly known as “Cedrón” and “Burrito”, respectively, are aromatic plant species known for their use in traditional medicinal and indigenous practice in South America, where they are native. *A. citriodora* is widely used in infusions for digestive disorders, in commercial beverages, insomnia management [18], and for its antioxidant activity [19], the study of its essential oil has demonstrated antibacterial and antifungal activity against phytopathogens such as *Alternaria linariae*, *Sclerotinia sclerotiorum*, and *Fusarium* sp. [20,21,22,23,24]. *A. polystachya* is one of the most frequently prescribed herbs in folk medicine, as an anxiolytic, in cardiovascular conditions [25,26,27,28] and for the treatment of the cultural-bound disease “empacho”, a digestive disorder [29,30]. Likewise, *A. polystachya* essential oil reports antibacterial activity [31], and regarding its antifungal activity, it shown to reduce the growth of *Fusarium verticillioides* (sin. *Fusarium moniliforme*), a species of fungus that infects corn [32]. The viability of *Aloysia* species as agricultural solutions is also reinforced by evidence of their safety profile. The traditional use of *A. citriodora* and *A. polystachya* in folk medicine, as well as toxicity studies conducted on *Artemia salina*, have shown that their essential oils are non-toxic [18]. At a regulatory level, the Food and Drug Administration (FDA) has classified *Aloysia citriodora* as a safe substance (GRAS), which allows for its incorporation into various food products [33]. However, to the best of our knowledge, no studies have determined the activities of the essential oils from the leaves of *A. polystachya* and *A. citriodora* on *Monilinia* sp. Therefore, the aim of this study was to determine the chemical composition and the antifungal efficacy of these essential oils and their components against the causal agents of postharvest rot: *M. fructicola*, *M. laxa*, and *B. cinerea*. Additionally, molecular docking of the most active compound (farnesol) was performed on the enzyme succinate dehydrogenase.

## 2. Results and Discussion

### 2.1. Chemical Composition of Essential Oils

Essential oil yield (weight/dry weight of plant) is 1.25% for *A. citriodora* leaves and 1.22% for *A. polystachya* leaves. GC-MS analysis of hydrodistillated *A. citriodora* essential oil allowed identifying 16 different components representing 99.93% of the relative proportion of these compounds in the oil. The global chromatographic analysis of *A. citriodora* oil showed a complex mixture consisting mainly of oxygenated mono- and sesquiterpenes and small amounts of sesquiterpene hydrocarbons. It was dominated by oxygenated sesquiterpenes (68.53%) and, to a lesser extent, by oxygenated monoterpenes (17.58%), while sesquiterpene hydrocarbons were present only in low percentages (13.82%). The major components detected in the oil were spathulenol (38.84%) and caryophyllene oxide (17.80%) followed by α-curcumene (8.57%), piperitone (7.22%), farnesol (5.13%), and citral (3.16%). In *A. polystachya* oil, GC/MS analysis identified 8 compounds, which represented 99.45% of the oil (Table 1). The major oil components were oxygenated monoterpenes, which usually occurs in the *Aloysia* genus; carvone was dominant among the major components (88.41%) followed by dihydrocarvone (4.57%) and *R*-limonene (3.90%).

Several studies have reported that the essential oil of *A. citriodora* typically contains high levels of monoterpenes [34,35,36]. In contrast, sesquiterpenes are usually present in moderate to low concentrations [18]. However, significant variations in the chemical composition of *A. citriodora* essential oils have been observed depending on geographic origin. Elechosa et al., studying the essential oils obtained from Northwestern Argentina, reported the existence of several chemotypes dominated by different components such as thujones (31.4–90.4%), citronellal (39.0–66.7%), carvone (48.2–70.9%), citral (51.0–54.3%), and linalool (78.2–85.1%) [37]. Similarly, in plants from the same region, Olmedo et al. found neral (27.3%), spathulenol (25.6%) and geranial (24.4%) as major constituents [38], getting closer to our results due to the higher proportion of spathulenol. In agreement with our findings, Oukerrou et al. reported a qualitatively similar sesquitepene-rich profile in *A. citriodora* cultivated in Morocco, with β-spathulenol (9.42–15.61%), α-curcumene (11.28–15.15%), and caryophyllene oxide (13.25–14.14%) as major components [20]. Along the same line, Tammal et al. identified spathulenol (13.07%) and caryophyllene oxide (11.78%) as predominant in oils extracted from plants collected in sub-humid regions of Tunisia [39]. In contrast, oils from arid and semi-arid zones in the same country were dominated by monoterpens particularly citral. Interestingly, Al-Maharik et al. reported that essential oils from *A. citriodora* grown in Jericho, an arid region in Palestine, exhibited a predominantly sesquiterpene profile (77.4%), with α-curcumene (26.94%), spathulenol (13.69%), geranial (10.79%), caryophyllene oxide (8.66%), and neral (7.59%) as the main constituents [40]. These findings suggest that the chemical composition of *A. citriodora* essential oils is highly variable and influenced by multiple factors such as regional climate, harvesting time, plant organ used, and the extraction method.

The chemical composition of the essential oil of *A. polystachya* was qualitatively consistent with that previously reported by our research group, in which the major constituents were *R*-carvone (91.03%), *R*-limonene (4.10%), and dihydrocarvone (1.07%) [41]. The predominance of *R*-carvone observed in this study aligns with reports from other *A. polystachya* oils obtained from Latin American regions, where *R*-carvone typically accounts for over 70% of the total composition [42,43].

### 2.2. Antifungal Activity of Essential Oils and Compounds

The essential oil of *A. citriodora* and some of its main constituents were active against *B. cinerea*, *M. fructicola*, and *M. laxa*, varying in levels of effectiveness. The *A. citriodora* oil showed the strongest inhibitory effect on the mycelial growth of *M. fructicola* (61.89 ± 1.16 µg/mL) and *M. laxa* (73.05 ± 3.1 µg/mL), being moderate for *B. cinerea* (183.26 ± 1.7 µg/mL), these results are shown in Table 2.

These findings are consistent with previous reports documenting the inhibitory activity of *A. citriodora* essential oil against phytopathogenic fungi such as *Alternaria linariae*, *Sclerotinia sclerotiorum*, and *Fusarium* sp. [22,24,39]. Similarly, other studies have demonstrated its moderate antifungal effect against *B. cinerea*, achieving 69% radial inhibition at a concentration of 250 ppm [44]. Fontana et al. also confirmed the oil’s efficacy against both *B. cinerea* and *M. fructicola*, reporting EC_50_ values of 0.63 mL/L and 0.21 mL/L, respectively [21]. This antifungal activity has often been attributed to its high content of monoterpenes, particularly citral and limonene, which are frequently described as the most abundant components in this species.

Among the analyzed components of the essential oil of *A. citriodora*, farnesol stood out for its potent antifungal activity. This compound registered the lowest EC_50_ values against *M. laxa* (45.32 ± 3.3 μg/mL) and *M. fructicola* (72.18 ± 0.07 μg/mL). It is important to note that there are no previous reports in the literature on the activity of farnesol specifically against *M. fructicola*. When comparing the results for *M. laxa* with those reported by Balsells-Llauradó et al. (2023), a superiority in potency is evident. Specifically, the maximum inhibition obtained against *M. laxa* was 84.2% at a concentration of 0.89 mg/mL [45]. This notable efficacy is achieved at a concentration up to 100 times lower than that used in the bibliographic study, establishing a considerably higher level of activity for pure farnesol against the tested strain. For the phytopathogen *B. cinerea*, an EC_50_ of 230.0 ± 3.1 μg/mL was determined. When contrasting this finding with the bibliography, it is evident that this concentration is significantly higher than the farnesol doses tested by Cotoras et al. (2012), whose maximum reported concentration was 166 μg/mL [46]. Given that our EC_50_ value exceeds the maximum concentration evaluated in that study by 1.38 times, this suggests a lower antifungal potency of farnesol under our assay conditions. In the present study, geraniol (EC_50_ = 163.4 ± 0.79 µg/mL) and nerolidol (EC_50_ = 173.5 ± 2.0 µg/mL) showed moderate activity against *M. fructicola* and *M. laxa*, respectively; however, their antifungal activity was notably lower against *B. cinerea*, with EC_50_ values exceeding 250 µg/mL for both. This low performance against *B. cinerea* partially contrasts with other reports in the literature, as one study calculated the EC_50_ of pure geraniol against this pathogen at 235 µg/mL, a value slightly better than the threshold observed here [47]. Additionally, it has been reported that geraniol can inhibit the mycelial growth of *B. cinerea* by 78% at a concentration of only 100 µg/mL and conidial germination by 96% at 250 µg/mL, suggesting that significant inhibition is possible at concentrations near this level [48]. On the other hand, the low activity of pure nerolidol against *B. cinerea* is well-supported, as despite being a main component (12.8%) of the essential oil of *Baccharis dracunculifolia*, one study showed that an extremely high concentration of 25 mg/mL was required to achieve 85.2% inhibition of mycelial growth. Therefore, the low performance observed in this work (EC_50_ > 250 µg/mL) is consistent with the characterization of nerolidol as a compound with moderate to low activity against this pathogen [49].

**Figure 1:**
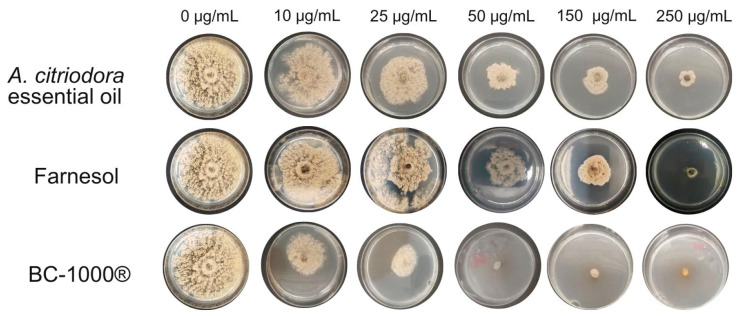
Inhibitory effect of *A. citriodora* essential oil, farnesol and BC-1000^®^ on the mycelial growth *of M. fructicola* according to different concentrations.

The inhibitory effects of most effective samples against *M. fructicola* are shown in Figure 1.

Nonetheless, several essential oils with sesquiterpene-rich profiles have also demonstrated strong antifungal properties. For example, the leaf essential oils of the medicinal plants *Lannea egregia* and *Emilia sonchifolia*, characterized by high concentrations of sesquiterpenes, were highly effective against *Aspergillus niger* [50]. Likewise, the fruit peel essential oil of *Hymenaea stigonocarpa*, in which spathulenol and caryophyllene oxide were the predominant compounds, showed antifungal activity against *Botrytis cinerea*, *Sclerotinia sclerotiorum*, and *Colletotrichum truncatum* [51]. Previous studies have shown that geraniol, nerolidol, and farnesol induce a cell death mechanism in filamentous phytopathogenic fungi by causing mitochondrial dysfunction and an increase in reactive oxygen species (ROS), leading to death by apoptosis [52,53,54]. In this study, the differences in the fungicidal activity of these compounds can be attributed to their respective chemical structures. Geraniol, being a monoterpene, is less lipophilic than farnesol, a sesquiterpene. This lower lipophilicity could limit its ability to penetrate fungal cell membranes. This hypothesis is supported by their logP values, with geraniol at 3.28, significantly lower than farnesol at 5.31. On the other hand, nerolidol and farnesol are both sesquiterpene alcohol isomers, which means they share the same molecular formula but differ in the spatial arrangement of their atoms. This characteristic is key, as farnesol is a primary allylic alcohol, making it more susceptible to oxidation than nerolidol, which is a tertiary alcohol. Additionally, farnesol longer chain and greater number of double bonds make it generally more prone to oxidation than geraniol [55]. The activity shown by farnesol is confirmed by a series of studies on phytopathogenic fungi such as *Fusarium graminearum*, *Penicillium expansum*, *Rhizoctonia solani* and *Ustilaginoidea virens* [46,56,57,58,59]. To the best of our knowledge, this is the first report of farnesol exhibiting inhibitory activity against species of the genus *Monilinia.* Despite belonging to the same genus, the essential oil from *A. polystachya* did not inhibit fungal growth, as none of its primary components—*R*-limonene, linalool, α-terpineol, dihydrocarvone, and *R*-carvone—showed significant activity. This can be attributed to its compositional profile, which differs from that of *A. citriodora* and lacks the specific bioactive metabolites effective against these filamentous fungi. This intraspecific variation is well-documented, as the efficacy of essential oils can differ based on their chemotype. For instance, studies on *A. polystachya* have shown that its carvone-rich chemotype is more effective against *B. cinerea* via fumigation than its α-thujone-rich chemotype [60]. Likewise, the main compound in *A. polystachya* oil, carvone, has been shown to inhibit the growth of filamentous fungi and even reduce the development of post-harvest lesions caused by *B. cinerea* in cherry tomatoes [61], also differing from our results. This discrepancy may be due to differences in the carvone isomer, the fungal strains, or the specific technique employed, as the aforementioned study applied carvone by fumigation it onto the PDA medium [61]. Regarding the existing evidence in *Monilinia* sp., our results are consistent with those obtained in other studies on dihydrocarvone, which followed the same methodology as our study, where this molecule has not been shown to inhibit mycelial growth and conidia germination of *Monilinia* sp. [62]. Consequently, *A. polystachya* does not appear to be a viable alternative for controlling these phytopathogens. Beyond efficacy, a critical consideration for any potential agricultural treatment is its impact on fruit quality. Previous research has shown that carvone, for instance, does not significantly affect the organoleptic quality of fruit upon application [61]. Similarly, other components found in essential oils, such as geraniol, nerolidol, and farnesol (present in the essential oil of *A. citriodora*), have been approved for foliar application in acaricide formulations like BioMite^®^, supporting their safety profile for agricultural use.

### 2.3. Molecular Docking

As a strategy to approach a possible mechanism of action of farnesol, a compound that has featured remarkably in antifungal assays, we have applied in silico assays on a target usually used by current chemical controls; the enzyme succinate dehydrogenase (SDH), also known as mitochondrial complex II, is an enzyme anchored to the inner mitochondrial membrane of aerobic organisms, which plays a key role in both the Krebs cycle and the electron transport chain. It is composed of four subunits: A and B, which are hydrophilic, and C and D, which are hydrophobic and embedded in the membrane [63].

Due to its metabolic relevance, SDH has been identified as a preferred target for the development of new fungicides [64,65,66]. Inhibitors of this enzyme constitute an important class of agricultural control agents, recognized for their high efficacy and, in some cases, broad-spectrum bactericidal activity [62]. The scientific literature describes a great structural diversity among potential SDH inhibitors; for example, Soto et al. [67] documented that certain hydrated geranylated phenol hydrates exert antifungal activity against *B. cinerea* through inhibition of this enzyme. In the present study, molecular docking of farnesol was performed using the crystal structure of SDH (PDB ID: 2FBW). The reference ligand was 2-methyl-N-phenyl-5,6-dihydro-1,4-oxathiine-3-carboxamide (CBE), present in the crystallized structure. The results (Table 3) show that farnesol exhibited a binding energy of −7.5 kcal/mol, slightly higher in affinity than CBE (−7.2 kcal/mol).

**Figure 2:**
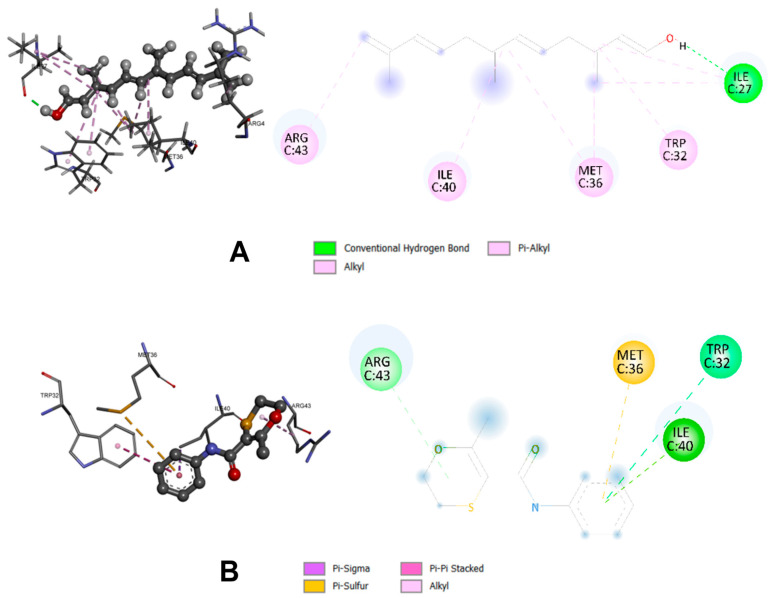
Three-dimensional and 2D interaction of farnesol (**A**) and CBE (**B**) into the allosteric site of enzyme SDH (PDB: 2FBW).

Both ligands shared interactions with key active-site residues, including Trp32, Met36, Ile40, and Arg43 (see Figure 2), suggesting that farnesol may bind to the same catalytic pocket and potentially interfere with enzymatic function in a comparable manner. Notably, farnesol also established an additional hydrogen bond with Ile27, absent in the CBE complex, which may contribute to increased complex stability and explain the modest improvement in calculated affinity. These findings are consistent with previous reports describing the antifungal activity of farnesol against *Candida albicans*, where it acts as a morphogenesis modulator, reduces biofilm formation, and enhances the effect of conventional antifungals [68,69]. In *B. cinerea*, it has been shown to inhibit mycelial growth and trigger an-apoptosis-like phenotype characterized by reactive oxygen species accumulation and structural damage [70].

Taken together, the observed affinity of farnesol for SDH, combined with a similar and partly superior interaction profile to that of a known inhibitor, supports the hypothesis that this sesquiterpene may act as a direct modulator of the enzyme. This highlights its potential as a structural scaffold for the design of new fungicides or as an adjuvant in the control of agriculturally important phytopathogens.

## 3. Materials and Methods

### 3.1. General Data

All reagents and compounds present of the essential oils were purchased from Sigma-Aldrich Co., (St. Louis, MO, USA), and AK Scientific Inc. (30023 Ahern Ave, Union City, CA 94587, USA).

### 3.2. Plant Material

Plant samples were collected, avoiding unhealthy specimens or signs of contamination, during spring of 2024, in Olmué, Valparaíso Region, Central Chile at an altitude of 200 mts (32°59′34″ S 71°09′34″ W) authentication was performed by Mr. Patricio Novoa, and voucher specimens (AC-0922 and AP-0922, *A. citriodora* and *A. polystachya*, respectively) was deposited for future reference in the Natural Products and Organic Synthesis Laboratory of Universidad de Playa Ancha, Valparaíso, Chile.

### 3.3. Essential Oil Extraction

To facilitate extraction, 500 g of each fresh plant was cut and ground in a food processor (Model: BLSTMGK15051, Oster, Racine, WI, USA). Each sample was then separately subjected to hydrodistillation with 2 L of water using a Clevenger-type apparatus [71]. The temperature was maintained at 100 °C until the water boiled, at which point it was reduced and controlled at 70 °C for 4 h. Hydrolate was subjected to liquid–liquid partition in a separator funnel and the obtained essentials oils were dried over anhydrous sodium sulfate and stored at 2–8 °C in amber glass vials. The yield of fresh essential oil was determined as the quotient of the weight of oil collected and the dry weight of plant material extracted. The yield was 1.25% for *A. citriodora* and 1.22% for *A. polystachya*.

### 3.4. Gas Chromatography Analyses

The sample (1 μL) was analyzed by Gas Chromatography–Mass Spectrometry (GC/MS). Analysis was carried out using a Hewlett-Packard GC/MS 6890 coupled to a Hewlett-Packard 5973 mass-selective detector (electron ionization, 70 eV, Palo Alto, CA, USA). Helium was used as carrier gas at a rate of 1.3 mL/min, and the capillary column used was a HP-5ms. The temperature program was 40 °C (5 min) to 280 °C (8 min) at a rate of 5 °C/min. Compounds in the chromatograms were identified by comparison of their mass spectra with those in the NIST 2021 library database, and by comparison of their retention index with those reported in the literature, for the same type of column or those of commercial standards, when available.

### 3.5. Antifungal Activity

#### Fungal Growth Conditions

Species used in this investigation were kindly provided by the collection of the Mycology Unit of the Servicio Agrícola y Ganadero (SAG) of Chile. The cultures were grown on 90 mm diameter Petri dishes on potato dextrose agar (PDA; DIFCO™).

### 3.6. Antifungal Assay In Vitro

Determination of mycelial growth inhibition of the samples was performed by measuring the radius of growth. The PDA media contained compounds or essential oils at concentrations between 10 and 250 µg/mL, previously dissolved in ethanol and water. The percentage of inhibition was determined according to methods described previously [72]. PDA 1% ethanol medium was used as a negative control, while a commercial fungicide, BC-1000^®^ (grapefruit extract of *Citrus x paridisi*, 50% *w*/*v*, CHEMIE, Providencia, Chile), was used as positive control and measured under the same conditions as the tested samples. For each treatment, a 4 mm disc of mycelium was inoculated in the center of a plate and incubated for 3 (for *B. cinerea*) o 5–7 days (*Monilinia* spp.), at 23 °C [62,73]. The results were expressed as the average effective concentration (EC_50_), that is, the concentration at which mycelial growth was reduced by 50%. This value was determined using Origin ProV. 8 software (OriginLab Corporation, Northampton, MA, USA). All treatments were performed in triplicate.

### 3.7. Molecular Docking

The three-dimensional models of the ligands were constructed using UCSF Chimera 1.18 software. Polar hydrogens were added to each ligand, Gasteiger charges were assigned, and energy minimization was performed using the General AMBER Force Field (GAFF). Graphical representations were created with the free version of Discovery Studio Visualizer. The crystal structure of succinate dehydrogenase (SDH, PDB ID: 2FBW, resolution 2.06 Å) was obtained from the Protein Data Bank http://www.rcsb.org/pdb (accessed on 11 August 2025). Molecular docking of SDH with the selected ligands was carried out using AutoDock4 software, applying the Lamarckian genetic algorithm. During docking, the protein was treated as rigid while ligands were fully flexible. Search parameters included 50 runs per ligand and a maximum of 25 million energy evaluations. A clustering threshold of RMSD < 0.5 Å was set. The cocristallized ligand in the enzyme, 2-methyl-N-phenyl-5,6-dihydro-1,4-oxathiine-3-carboxamide (CBE), was used as a reference for docking. The optimal protein–ligand complex was selected based on the most populated cluster with the lowest binding free energy (ΔG). To validate the accuracy of the protocol, the cocristallized ligand was redocked under the same conditions, yielding an RMSD of 1.29 Å. All experiments were conducted at physiological pH.

## 4. Conclusions

This study determined the composition and evaluated the in vitro antifungal activity of the essential oils from *A. citriodora* and *A. polystachya*. The results revealed significant differences in both the types of molecules present and their respective efficacy. The oil from *A. citriodora*, rich in oxygenated sesquiterpenes, showed greater activity against *M. fructicola* and *M. laxa* compared to the *A. polystachya* oil, which is a mixture primarily composed of oxygenated monoterpenes. This difference suggests a possible synergistic effect among the constituents of the *A. citriodora* oil. Of the compounds evaluated from both species, farnesol, found exclusively in *A. citriodora*, demonstrated strong antifungal activity against the same pathogens, unlike the bioactive metabolites present in *A. polystachya*. Based on these findings, the essential oil of *A. citriodora* and farnesol, in particular, show great potential as antifungal agents. However, further studies are needed to develop stable formulations that overcome the inherent instability of essential oils in practical applications.

## Figures and Tables

**Table 1:** Chemical composition of *Aloysia citriodora* and *Aloysia polystachya* essential oil.

No	Rt (min)	Compound	RI ^a^	RL ^b^	*A. citriodora*	*A.* *polystachya*	Identification
					*% Area*	*% Area*	
1	11.10	*R*-limonene	1031	1031		3.90	RL, MS, Co
2	13.16	Linalool	1107	1107		0.78	RL, MS, Co
3	13.75	*trans*-*p*-mentha-2,8-dienol	1113	1113		0.48	RL, MS
4	14.73	8,9-dehydrothymol	1120	1121	1.72		RL, MS
5	15.11	Nerol	1205	1205	1.48		RL, MS, Co
6	15.55	Neral	1209	1209	1.64		RL, MS, Co
7	15.82	α-terpineol	1160	1160		0.48	RL, MS, Co
8	15.97	Geraniol	1232	1232	2.36		RL, MS, Co
9	15.98	dihydrocarvone	1200	1200		4.57	RL, MS, Co
10	16.53	Citral	1241	1241	3.16		RL, MS, Co
11	17.31	*R*-carvone	1242	1242		88.41	RL, MS, Co
12	18.91	Piperitenone	124	1243	7.22		RL, MS
13	22.04	caryophyllene	1418	1418		0.62	RL, MS
14	23.29	α-curcumene	1482	1483	8.57	0.21	RL, MS
15	23.81	bicyclogermacrene	1474	1475	2.25		RL, MS
16	24.35	Cubebol	1510	1511	2.07		RL, MS
17	25.65	Nerolidol	1548	1548	2.11		RL, MS, Co
18	26.21	Spathulenol	1572	1572	38.84		RL, MS
19	26.39	caryophyllene oxide	1578	1578	17.80		RL, MS
20	26.54	*t*-cadinol	1636	1638	1.90		RL, MS
21	27.98	Z-nuciferol	1699	1700	1.07		RL, MS
22	28.40	*E*-nuciferol	1725	1726	2.61		RL, MS
23	28.45	Farnesol	1730	1730	5.13		RL, MS, Co
Monoterpene hydrocarbons						3.9	
Oxygenated monoterpenes Sesquiterpene hydrocarbons					17.58	94.72	
					13.82	0.83	
Oxygenated sesquiterpenes					68.53		
Total					99.93	99.45	

^a^ Experimental retention index; ^b^: bibliographic retention index, MS: Mass spectra, Co: co-elution with standard compounds available in our laboratory.

**Table 2:** EC_50_ values (µg/mL) of *A. citriodora* and *A. polystachya* essential oil and their compounds on the in vitro mycelial growth inhibition of *B. cinerea*, *M. fructicola* and *M. laxa*.

Sample	*B. cinerea* ^a^	*M. fructicola* ^a^	*M. laxa* ^a^
*A. citriodora* essential oil	183.26 ± 1.7 ^b^	61.89 ± 1.16 ^b^	73.05 ± 3.1 ^c^
Citral	>250	>250	>250
Nerol	>250	>250	>250
Nerolidol	>250	>250	173.5 ± 2.0 ^d^
Gerianol	>250	163.4 ± 0.79 ^d^	>250
Farnesol	230.0 ± 3.1 ^c^	72.18 ± 0.07 ^c^	45.32 ± 3.3 ^b^
*A. polystachya* essential oil	>250	>250	>250
*R*-limonene	>250	>250	>250
Linalool	>250	>250	>250
α-terpineol	>250	>250	>250
Dihydrocarvone	>250	>250	>250
*R*-carvone	>250	>250	>250
BC-1000^®^	45.97 ± 2.7 ^a^	10.55 ± 1.74 ^a^	13.64 ± 1.3 ^a^

^a^ EC_50_ values are expressed as the mean ± SD of experiments performed in triplicate. Superscript letters indicate significant differences between treatments according to the LSD multiple comparison test (*p* < 0.05) (only between active compounds).

**Table 3:** Binding energy and interactions of farnesol and CBE with SDH.

Ligand	Binding Energy [kcal/mol]	Interactions
Farnesol	−7.5	Ile27; Trp32; Met36; Ile40; Arg43
CBE	−7.2	Arg43; Met36; Trp32; Ile40

## Data Availability

All data are available for scientific community on the manuscript.
